# Antimicrobial and Synergistic Activity of 2,2′,4-Trihydroxybenzophenone Against Bacterial Pathogens of Poultry

**DOI:** 10.3389/fmicb.2019.00490

**Published:** 2019-03-20

**Authors:** Martha Isabel Realpe Aranda, Gabriel Andres Tafur Gómez, Mariana de Barros, Marcelo Henrique dos Santos, Leandro Licursi de Oliveira, Junnia Luisa Pena, Maria Aparecida Scatamburlo Moreira

**Affiliations:** ^1^Departamento de Veterinária, Laboratório de Doenças Bacterianas, Universidade Federal de Viçosa, Viçosa, Brazil; ^2^Universidad de Ciencias Aplicadas y Ambientales, Bogotá, Colombia; ^3^Departamento de Química, Laboratório de Síntese de Agroquímicos, Universidade Federal de Viçosa, Viçosa, Brazil; ^4^Departamento de Biologia Geral, Laboratório de Imunoquímica e Glicobiologia, Universidade Federal de Viçosa, Viçosa, Brazil

**Keywords:** poultry diseases, benzophenone, antimicrobial agents, drug synergisms, bactericide

## Abstract

In poultry farming, the spread of bacterial pathogens results in disease outbreaks causing significant economic losses to this industry. Many of these pathogenic bacteria are zoonotic and have a substantial impact on public health. Antimicrobials are essential for the prevention and treatment of these bacterial infections. However, the indiscriminate use of these agents provides favorable conditions for selection, propagation and persistence of bacteria and development of antimicrobial resistance. We developed a new antimicrobial candidate that could be used alone or in synergy with research protocols for therapeutic, prophylactic and growth promoter uses in the poultry industry. The present study aimed at evaluating the antimicrobial activity of the synthetic compound 2,2′,4-trihydroxybenzophenone against pathogenic bacteria that cause important diseases in poultry and public health. We tested the hemolytic effect of this compound, studied its synergistic effect with conventional antimicrobials and analyzed the site of action on the bacteria. The results of our study showed antimicrobial activity of benzophenone against Gram-positive and Gram-negative bacteria with a similar effect in ATCC (American type culture collection) and field isolates. This compound was non-hemolytic. 2,2′,4-trihydroxybenzophenone acted on the bacterial cell wall. We identified the synergistic effect between 2,2′,4-trihydroxybenzophenone and bacitracin, this effect indicate that antimicrobial synergism may be useful for the treatment of necrotic enteritis in poultry. This compound may also be used as a growth promoter by reducing the dose of bacitracin and thus decreasing the pressure of bacterial resistance in poultry which would circumvent the development of cross-resistance in humans.

## Introduction

Poultry farming is the most globalized industry in terms of food production worldwide. Considering the low costs of production, chicken meat, and eggs are major food sources for rapidly growing populations. In addition, there are no constraints or restrictions imposed by any religion on the consumption of these types of food (Landoni and Albarellos, 2015). The rapid technological advances and competitiveness in the poultry industry in recent decades have imposed extreme conditions for animal health. Current production systems are based on high animal density which provides ideal conditions for the multiplication, propagation, and spread of pathogens and the occurrence of disease outbreaks resulting in significant losses to the poultry industry.

Among these pathogens, there are those related to public health which further extends the importance of prevention and control of foodborne zoonotic bacteria that may occur in poultry products (Garcia-Migura et al., 2014). In addition, the unrestrained use of antimicrobials for the control and treatment of bacterial diseases and as growth promoters provide favorable conditions for the selection, propagation, and persistence of antibiotic-resistant bacteria in both animals and humans (Aidara-Kane et al., 2018). The more intense use of antimicrobial agents in the poultry industry favors the increase of multidrug resistant bacteria *Escherichia coli*, *Salmonella*, *Pasteurella multocida*, *Clostridium perfringens*, and *Staphylococcus aureus* (Charlebois et al., 2012; Garcia-Migura et al., 2014; Victor et al., 2016; Kraushaar et al., 2017).

Similarly, poultry is considered an important reservoir of bacteria *Salmonella* Typhimurium and *S. aureus* (Mughini-Gras et al., 2014; Löfström et al., 2015). These two species of bacteria have a major impact on human and animal health and may cause significant economic losses (Ailes et al., 2013; Peton and Le Loir, 2014). *S.* Typhimurium was the bacteria most often cultured from cases of salmonellosis in humans in Europe (European Food Safety Authority [EFSA] and European Centre for Disease Prevention and Control [ECDPC], 2013). These microorganisms were associated with multiple foodborne outbreaks in Australia which were linked to the consumption of eggs (Moffatt et al., 2016) or chicken meat (Fearnley et al., 2011). *S. aureus* cultured from poultry samples was phylogenetically identical to the microorganism cultured from human specimens which draw attention to the possibility of the transmission of this bacterium between poultry and humans and the zoonotic potential of this pathogen (Lowder et al., 2009). In the same way, the number of isolates of *S. aureus* with antimicrobial resistance profiles have been reported in several countries (Kraushaar et al., 2017).

The poultry industry is affected with *C. perfringens* that causes necrotic enteritis in chickens which is a disease of increasing importance to the poultry industry, resulting in a total cost worldwide of more than $6 billion per year due to disease outbreaks (Wade et al., 2015). Bacitracin is one of the most common antimicrobial agents used to control this bacterial enteric avian disease and has also been used as a performance enhancer in poultry in several countries (Reis et al., 2014; Han et al., 2015). However, the extensive use of bacitracin appears to have led to the isolation of bacitracin-resistant *C. perfringens* strain (Charlebois et al., 2012).

Antimicrobial therapy is the most commonly used method for the treatment of diseases caused by resistant bacteria. Therefore, there is a great demand and interest in the search for alternative antimicrobial molecules. Natural benzophenones are an important alternative for antimicrobial use which are effective against Gram-positive and Gram-negative bacteria, and are not cytotoxic for mammalian cells. Based on the results of bacterial inhibition obtained with other derivatives of natural benzophenones and in the search for new antimicrobial molecules, we present the first study to evaluate the antimicrobial activity of the synthetic compound 2,2′,4-trihydroxybenzophenone against a large panel of ATCC bacteria and bacterial field isolates of significance in the poultry industry and public health. We assessed the cytotoxic effect of benzophenone on chicken erythrocytes, its synergistic effect with antimicrobials commonly used in poultry farming, especially with bacitracin, and the site of action of this compound on the pathogenic bacteria.

## Materials and Methods

### Substances Used

2,2′,4-Trihydroxybenzophenone (PubChem CID: 578663) is a derivative of benzophenone which is a synthetic molecule, its physico-chemical characteristics are described below: empirical formula: C_13_H_10_O_4_; molar mass: 230.21 u.m.a.; log P: 3.46; donor groups (NH + OH): 3; acceptor groups (N + O): 4 (Doriguetto et al., 2007).

The conventional antimicrobials used are representative of the main classes used in poultry farming including bacitracin (INLAB, São Paulo, Brazil), lincomycin (GENFAR, Bogotá, Colombia), amoxicillin (Mk Cali, Colombia) and erythromycin (Mk Cali, Colombia).

### Bacterial Isolates and Inocula

We included bacterial species that cause diseases in poultry and are important in terms of public health. We selected the Gram-positive bacteria *C. perfringens* ATCC 12924, *S. aureus* ATCC 27659 and *S. aureus* field isolate. The Gram-negative bacteria selected were *Escherichia coli* ATCC 25922, *P. multocida* subsp. *multocida* ATCC 6530, *Salmonella enterica* subsp. *enterica* serovar Enteritidis ATCC 13076 and *S. enterica* subsp. *enterica* serovar Typhimurium ATCC 13311, *S. enterica* subsp. *enterica* serovar Gallinarum ATCC 9184, *S. enterica* subsp. *enterica* serovar Pullorum ATCC 9120. In addition, field isolates of *E. coli*, *P. multocida* subsp. *multocida*, *S. enterica* subsp. *enterica* serovar Enteritidis and *S. enterica* subsp. *enterica* serovar Typhimurium were used.

The source of the bacterial isolate ATCC was Fundação Oswaldo Cruz, Rio de Janeiro – RJ – Brazil. The source of field isolate was Universidade Federal de Minas Gerais, Belo Horizonte – MG – Brazil and Mercolab, Cascavel – PR – Brazil. Initially, 50 μL of each isolate from the bacterial stock was activated in 3 mL of Tryptic Soy Broth (TSB) (Himedia) and kept in an incubator at 37°C for 24 h to stimulate bacterial multiplication. After that, each isolate was streaked on Tryptic Soy Agar (TSA) (Himedia) plates and maintained for 24 h in an incubator at 37°C. Three colonies were resuspended in 3 mL of TSB broth and their concentrations were adjusted to a standard of 0.5 McFarland, the approximate equivalence of 1–2 × 10^8^ CFU/mL, after counting. Samples were then diluted 1:10,000 in TSB to obtain 1 × 10^4^ CFU/mL.

### Determination of Minimum Inhibitory Concentration (MIC) and Minimum Bactericidal Concentration (MBC)

The broth microdilution method was used to determine the MIC values according to the Clinical and Laboratory Standards Institute [CLSI] (2012) with minor modifications. For this purpose, 1 mg of benzophenone was diluted in 50 μL of dimethylsulfoxide DMSO (Merck) followed by the addition of 950 μL of PBS to a final concentration of 1 mg/mL in 5% of DMSO. To obtain the different concentrations, a serial dilution of 1:2 was carried out in PBS, yielding the concentrations of 250, 125, 62.50, 31.25, 15.62, and 7.81 μg/mL. Then, each dilution was inoculated in wells of a microtiter plate (Sarstedt 42192); 100 μL of the inoculum of each bacterium (1 × 10^4^ CFU/mL) was added to each well plate. Bacterial suspensions in the TSB medium were used as positive controls, and TSB medium sterile was used as the negative control. Plates were incubated at 37°C for 24 h. Microplates were read on a spectrophotometer (Thermo Fisher Scientific, Multiskan FC) with a wavelength of 450_nm_. The MIC was considered as the lowest concentration capable of preventing bacterial multiplication, compared with the controls. The MIC values were obtained by calculating the means of the results.

The percentage of bacterial survival was calculated as follows:

$$\left[\frac{\left(\mathrm{mean optical density of sample}-\mathrm{mean optical density of negative control}\right)}{\left(\mathrm{mean optical density of control}-\mathrm{mean optical density of negative control}\right)}\right]\times 100\%$$

After the plates were read on a spectrophotometer, wells of the dilutions with visible bacterial multiplication were identified; 10 μL of bacteria were streaked on TSA plates for 24 h at 37°C. Subsequently, colonies were counted to determine the MBC which was considered the lowest concentration where there was no formation of colonies. All MIC and MBC assays were performed in three independent experiments with three repetitions per experiment.

### Site of Action of Benzophenone – Bacterial Wall

To evaluate the effect of benzophenone on the bacterial wall, three methodologies described below were used. *S.* Typhimurium and *S. aureus* field isolates were used as Gram-negative and Gram-positive bacterial models, respectively. The working concentration of the bacterial inoculum was 1 × 10^8^ CFU/mL. The concentration of 2,2′,4-trihydroxybenzophenone was 16 × MIC to make bacterial wall effects more evident, corresponding to 1 mg/mL for *S. aureus* and 2 mg/mL for *S.* Typhimurium.

#### Protein Release – Bradford Assay

Each bacterial suspension was treated with benzophenone for 1, 2, and 3 h. Then these suspensions were centrifuged at 4°C for 30 min at 300 × *g*. A supernatant was obtained in which protein release was evaluated using the Bradford method (Bradford, 1976). The determination of the protein concentration was calculated based on the standard curve performed with bovine serum albumin (BSA) at a O.D. of 595_nm_. All assays were performed in three independent experiments with three replicates per experiment.

#### Flow Cytometry

We used the protocol published by Coronel-León et al. (2016) with minor modifications. The procedure was performed by adding 100 μL of the benzophenone solution plus 100 μL of the bacterial inoculum which was incubated at 37°C for 1 and 2 h. The bacterial inoculum without benzophenone was used as the negative control. Samples were then stained with propidium iodide (PI) in the dark. Readings were performed using the flow cytometer (BD FACS Verse) at an excitation wavelength of 488_nm_.

#### Transmission Electron Microscopy (TEM)

The protocol used was the one published by Nacif-Marçal et al. (2015). Fifty micro liter of the benzophenone solution and 50 μL of the bacterial inoculum were added to a microtube and then incubated at 37°C for 60, 120, and 180 min. A bacterial inoculum without benzophenone was used as the negative control. After incubation, cultures were centrifuged for 10 min at 1400 × *g*, and the pellet was resuspended with 50 μL of PBS; 7 μL of the bacterial solution was placed on a grid with the addition of 2% uracil for 15 s and was allowed to dry for 24 h. Then samples were examined under a transmission electron microscope (Zeiss EM 109).

### Evaluation of Hemolytic Activity

This study and the research proposal were approved by the Animal Research Ethics Committee of the university (CEUA/UFV), permit 47/2015. The hemolytic activity of benzophenone was assessed using erythrocytes of *Gallus gallus domesticus* according to the protocol published by Yacoub et al. (2016). Three 40 week-old healthy chickens weighing approximately 2.5 kg were used; 3 mL of blood were sampled from each layer hen. Blood specimens were collected by ulnar venipuncture using tubes with heparin. Whole blood was centrifuged at 1500 × *g* for 10 min at 20°C. Plasma was discarded in order to obtain erythrocytes. After three washes with PBS, these erythrocytes were used in the test. 100 μL was added to each well of the microtiter plate (Sarstedt 42192) along with 100 μL of the same concentrations of benzophenone used for the MIC (250, 125, 62.50, 31.25, 15.62, and 7.81 μg/mL, DMSO control and positive control). Then the plate was incubated at 37°C for 2 h. The plate was then centrifuged at 800 × *g* for 10 min. The supernatants were transferred to a new microtiter plate to analyze the release of hemoglobin in the spectrophotometer (Thermo Fisher Scientific, Multiskan FC) with a wavelength of 405_nm_. All hemolysis assays were performed in three independent experiments with three repetitions per experiment. Controls for 0 and 100% hemolysis consisted of erythrocytes suspended in PBS and 1% Triton X-100, respectively. The percentage of hemolysis was calculated as follows:

$$\left[\frac{\left(A\;405\mathrm{nm},\;\mathrm{benzophenone}-A\;405\mathrm{nm},\;\mathrm{PBS}\right)}{(A\;405\mathrm{nm},1\%\;\mathrm{Triton}\;X\;100-A\;405\mathrm{nm},\;\mathrm{PBS}}\right]\times 100\%$$

### Synergism

After identifying toward which traditional antibiotics the bacteria selected for this study had low sensitivity, these antimicrobials were used in the benzophenone synergism assay in serial dilutions of 2 × MIC. The Checkerboard method was used according to Brahim et al. (2015) with minor modifications; 50 μL of said antimicrobials were placed along each well of the ordinates of the 96 well microtiter plate and the benzophenone dilutions were arranged along the abscissa (50 μL). Then, 100 μL of the bacterial suspension (1 × 10^4^ CFU/mL) was added to each well. Plates were incubated at 37°C for 24 h. All assays were performed in triplicate and repeated three times. Results were evaluated algebraically by the fractional inhibitory concentration index (FlCi) according to the following equation:

$$\text{FICi}=\frac{\mathrm{MICab}}{\mathrm{MICa}}+\frac{\mathrm{MICab}}{\mathrm{MICb}}$$

MICa = MIC of antimicrobial alone; MICb = MIC of benzophenone alone and MICab is the MIC of antimicrobial in combination with benzophenone.

The interpretation of results will be: total synergism (FICi ≤ 0.5), partial synergism (0.5 < FICi ≤ 0.75), no synergism (0.75 < FICi ≤ 2), or antagonism (FICi > 2), the isobolograms were prepared according to Hewlett (1969).

### Statistical Analysis

Data were analyzed obtaining the mean value and the standard mean deviation. The averages obtained from the MIC test and protein release assay were compared by *F*-test. The significance level was 5% (*p* < 0.05). All statistical analyses were performed using software SAS version 9.3 (Statistical Analysis System 9.3, 2012, SAS Institute Inc.) licensed by the Federal University of Viçosa (UFV).

## Results

### Antimicrobial Activity – MIC and MBC

Minimum inhibitory concentration values of benzophenone for the bacteria tested ranged between 62.5 and 250 μg/mL, and MBC ranged from 125 to 500 μg/mL (Table 1 and Supplementary Figure S1). Statistical analysis showed that there was no significant difference in the antimicrobial activity of benzophenone against ATCC bacteria and bacterial field isolates.

**Table 1:** Minimum inhibitory concentration (MIC) and minimum bactericidal concentration (MBC) of 2,2′,4-trihydroxybenzophenone compound against ATCC bacteria and field isolates that affect poultry and are of major public health concern.

Classification	Specie	MIC		MBC
			(μg/mL)	
Gram-negative	*Escherichia coli*^∗^	125		250
	*E. coli* ATCC 25922	125		250
	*Pasteurella multocida* subsp. *multocida*^∗^	62.5		125
	*P. multocida* subsp. *multocida* ATCC 6530	62.5		125
	*Salmonella enterica* subsp. *enterica* serovar Enteritidis^∗^	250		500
	*S. enterica* subsp. *enterica* serovar Enteritidis ATCC 13076	250		500
	*S. enterica* subsp. *enterica* serovar Gallinarum ATCC 9184	125		250
	*S. enterica* subsp. *enterica* serovar Pullorum ATCC 9120	250		500
	*S. enterica* subsp. *enterica* serovar Typhimurium^∗^	125		250
	*S. enterica* subsp. *enterica* serovar Typhimurium ATCC 13311	62.5		125
Gram-positive	*Staphylococcus aureus* ATCC 27659	62.5		125
	*S. aureus^∗^*	62.5		125
	*Clostridium perfringens* ATCC 12924	125		250

*^∗^Field isolates.*

### Action on Bacterial Wall

**FIGURE 1:**
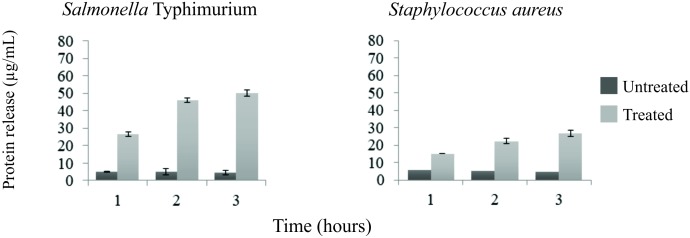
Protein release in the culture supernatant of treated and untread (controls) *Salmonella* Typhimurium and *Staphylococcus aureus* with 2,2′,4-trihydroxybenzophenone (2 and 1 mg/mL, respectively) evaluated by Bradford method. Each bar represents the mean value, the standard mean deviation is presented in the error bar. There was difference between treated and untreatead bacteria, by *F*-test, at 5% of significance.

**FIGURE 2:**
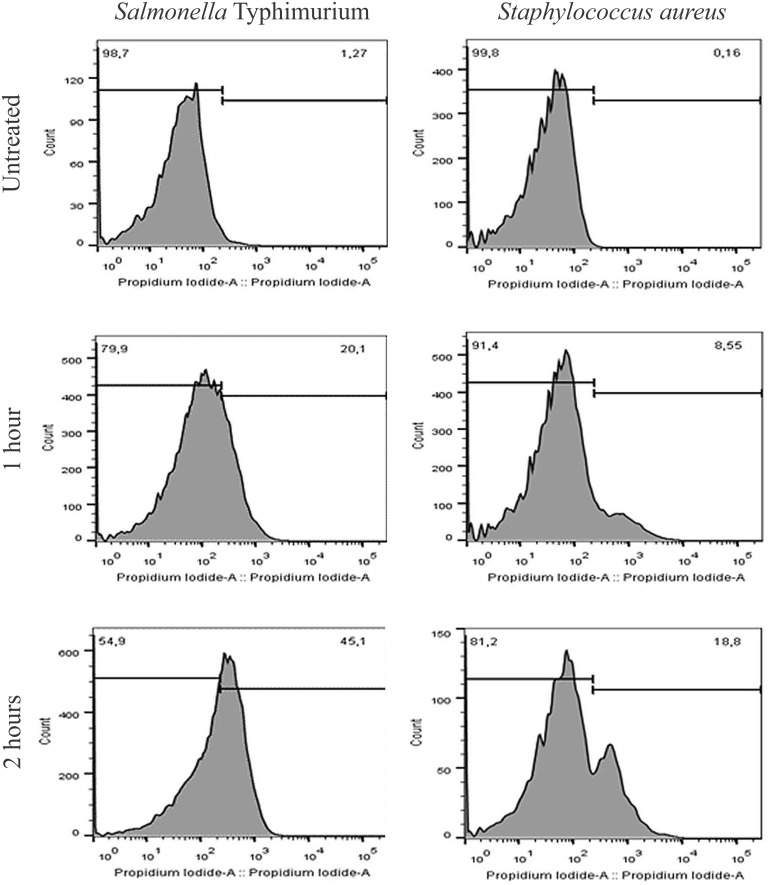
Flow cytometric evaluation of subpopulations of the *Salmonella* Typhimurium and *S. aureus* after treatment with 2,2′,4-trihydroxybenzophenone (2 and 1 mg/mL, respectively) by 1 and 2 h. Each peak represents a subpopulation and the numbers represent the percentage of cells of each one.

**FIGURE 3:**
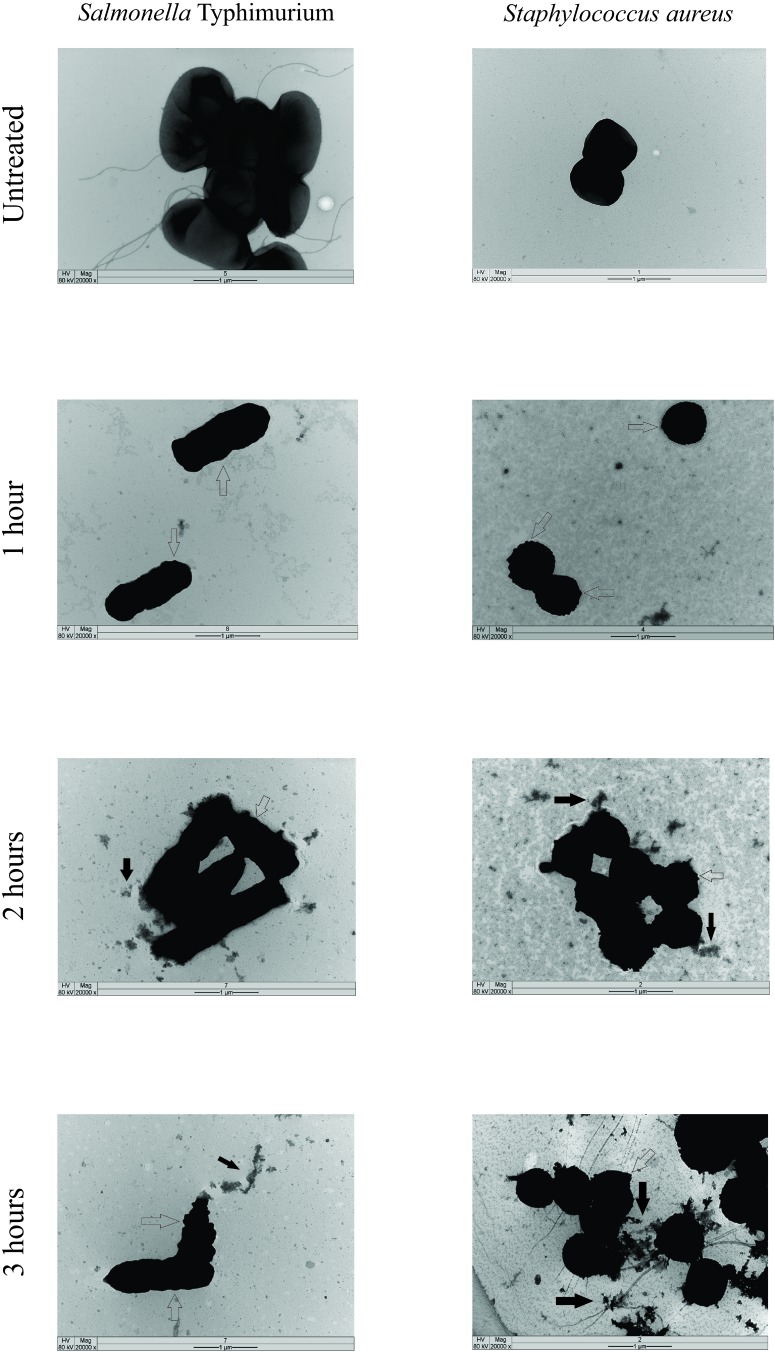
Transmission electron microscopy images of treated and untreated *Salmonella* Typhimurium and *S. aureus* with 2,2′,4-trihydroxybenzophenone (2 and 1 mg/mL, respectively). Empty arrows point to the morphological changes in the cell wall and full arrows point to the extravasation of cytoplasmic content. Scale bar: 1 *μ*m.

Release of proteins was observed in both Gram-positive and Gram-negative bacteria. Leakage of proteins from benzophenone-treated bacteria was significantly higher in comparison with the control group (*p* < 0.05) (Figure 1). Two subpopulations of bacterial cells were observed by flow cytometry: a subpopulation of intact cells and a subpopulation stained with PI, indicating cell permeability leading to death of these cells. The progression of bacterial death was increased according to the contact time of benzophenone with field bacteria reaching 45.1% after 2 h for *S.* Typhimurium and 18.8% after 2 h for *S. aureus* (Figure 2). When bacteria were exposed to benzophenone for 1 h, morphological changes were observed in the bacterial wall including small wrinkles and a rough and irregular outer surface (Figure 3). When the time span was 2 and 3 h, the bacterial wall was severely damaged and showed severe morphological changes resulting in leakage of cytoplasmic contents to the extracellular medium (Figure 3).

### Hemolytic Activity

The hemolytic activity was evaluated using the freshly isolated chicken erythrocytes that were incubated with 2,2′,4-trihydroxybenzophenone. We observed that even when the highest concentration obtained in the MIC (250 μg/mL) was used, there was only 2.56% of hemolysis compared with the positive control. In the other concentrations, hemolysis was below this value or there was no hemolytic activity. This fact was also noted for the DMSO (5%) used in the dilution of benzophenone (Table 2).

**Table 2:** Hemolysis of laying hens erythrocytes after treatment for 2 h with different concentrations of 2,2′,4-trihydroxybenzophenone.

2,2′,4-trihydroxybenzophenone concentrations (μg/mL)	Hemolysis (%)
C+	100
C−	0
DMSO	0.2
7.81	0.25
15.6	0.28
31.25	0.34
62.5	0.79
125	0.88
250	2.56

*The percentage values represent the hemolysis of the cells compared to the controls. C+: positive control, 100% hemolysis (erythrocytes with Triton X-100 1%). C−: negative control, 0% hemolysis (erythrocytes in phosphate buffered saline). DMSO: control of the diluent dimethylsulfoxide (5%).*

### Synergism

**FIGURE 4:**
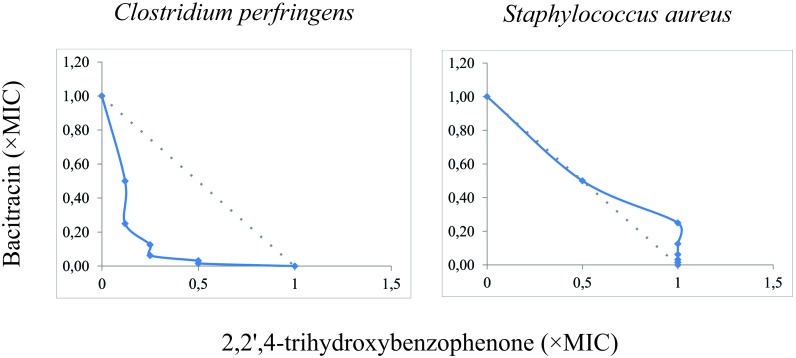
Isobolograms of the interaction between 2,2′,4-trihydroxybenzophenone and bacitracin for *Clostridium perfringens* ATCC 12924 and *S. aureus* ATCC 27659. Gray dotted line: FICI = 1.

According to the results of the MICs presented in Table 3, *C. perfringens* ATCC 12924 and *S. aureus* ATCC 27659 showed low sensitivity only for bacitracin. FICi showed that there was synergism between 2,2′,4-trihydroxybenzophenone and bacitracin against *C. perfringens* ATCC 12924. No synergism was observed in *S. aureus* ATCC 27659 (Figure 4 and Table 4).

**Table 3:** Minimum inhibitory concentration (MIC) of antimicrobials used in treatment of pathogenic bacteria ATCC, source: Fiocruz, Rio de Janeiro, Brazil.

Bacteria	Antibiotic	MIC (μg/mL)
*C. perfringens* ATCC 12924	Bacitracin	125
	Lincomycin	0.98
	Amoxicillin	15.62
	Erythromycin	3.95
*S. aureus* ATCC 27659	Bacitracin	62.5
	Lincomycin	1.97
	Amoxicillin	0.98
	Erythromycin	0.49
*S. enterica* subsp. *enterica* serovar Typhimurium ATCC 13311	Lincomycin	1.97
	Amoxicillin	3.95
	Erythromycin	0.49

*ATCC, American Type Culture Collection.*

**Table 4:** Interactions of benzophenone with bacitracin in ATCC bacteria.

Bacteria	Antibiotic	MIC		FICi	Effect
		Alone	Combined		
*C. perfringens* ATCC 12924	Benzophenone Bacitracin	125 125	7.8 31.25	0.31	Synergism
*S. aureus* ATCC 27659	Benzophenone Bacitracin	62.5 62.5	7.8 62.5	1.12	No synergism

*ATCC, American type culture collection. MIC, minimum inhibitory concentration. FICi, fractional index of inhibitory concentration=*$\frac{\mathrm{MICab}}{\mathrm{MICa}}+\frac{\mathrm{MICab}}{\mathrm{MICb}}$*, where MICa = MIC of antimicrobial alone; MICb = MIC of benzophenone alone and MICab is the MIC of antimicrobial in combination with benzophenone.*

## Discussion

The synthetic molecule 2,2′,4-trihydroxybenzophenone was, for the first time, evaluated here against Gram-positive and Gram-negative bacteria that affects poultry and cause concerns on public health.

Based on MIC and MBC, our findings show that 2,2′,4-trihydroxybenzophenone is a promising antibacterial candidate, as it was shown that has antimicrobial activity against the Gram-positive and Gram-negative bacterial strains tested. Besides, this compound demonstrated similar effect in both ATCC bacteria and avian field isolates what is a significant finding, since field isolates are usually subjected to selective pressures resulting from the inadequate administration of conventional antimicrobials (Table 1). The antimicrobial effect may be related to the presence of hydroxyl groups in 2,2′,4-trihydroxybenzophenone in the same way as in xanthones, which are natural derivatives of benzophenone with hydroxyl groups that have high antibacterial activity (Dharmaratne et al., 2013; Koh et al., 2015).

In our study, *S.* Typhimurium and *S. aureus* were used as models to evaluate the action of benzophenone on the bacterial wall once these bacteria present different wall structures (European Food Safety Authority [EFSA] and European Centre for Disease Prevention and Control [ECDPC], 2013), besides having a huge impact on human and animal health, as well as inducing economic losses (Ailes et al., 2013; Peton and Le Loir, 2014). The effect of benzophenone on these bacterial walls was assessed by three different methods. Using the Bradford method (Bradford, 1976), it was demonstrated that there was leakage of the intracellular proteins from both *S.* Typhimurium and *S. aureus* when in contact with benzophenone (Figure 1). Benzophenones might target the bacterial membrane resulting in its depolarization (Vooturi et al., 2009) and already showed affinities to polyanionic components of the cell wall, such as lipoteichoic acid and lipopolysaccharide, through their cationic residues (Vooturi et al., 2011). The permeability of the bacterial wall was also evaluated by flow cytometry, we observed a time-dependent effect in both Gram-negative and Gram-positive bacteria when in contact with benzophenone (Figure 2). However, in the thin wall of Gram-negative bacteria the action was faster compared with the thick Gram-positive bacteria wall, which suggests that the action of 2,2′,4-trihydroxybenzophenone is influenced by the composition of peptidoglycans (Yount and Yeaman, 2013). The morphology was studied by transmission electron microscopy (TEM) that showed alteration in the appearance of bacteria with the formation of roughness in the cell wall and extravasation of cytoplasmic content with the increase of contact time with 2,2′,4-trihydroxybenzophenone (Figure 3). This pleomorphic appearance can be related to changes in the cytoskeleton of the bacteria that would compromise the integrity of the bacterial wall (Wang et al., 2015), once occurs protein extravasation after 2,2′,4-trihydroxybenzophenone exposition what would damage bacterial wall as natural consequences of antimicrobial activity of this molecule.

Evaluating toxicity to avian cells, we used erythrocytes of chickens, since hemolysis is one of the main side effects caused by membrane bound antibiotics (Vooturi et al., 2011). Apart from the significant antimicrobial activity of 2,2′,4-trihydroxybenzophenone, this compound displayed low hemolytic activity even in the highest concentration tested (250 μg/mL). Previously it has been shown that benzophenones can disrupt the lipid vesicles mimicking the lipid compositions of Gram-negative and Gram-positive cell membranes but not induce alteration in lipid vesicles of animal cell membrane (Vooturi et al., 2011). This property may be attributed to the higher levels of cholesterol in the membranes of the animal cells and to the lack of negatively charged molecules incorporated into the phospholipid bilayer of animal membranes, which would contribute to the protection of erythrocytes (Matsuzaki et al., 1995). Thus, 2,2′,4-trihydroxybenzophenone selectively acts on bacterial membranes and therefore can be relied upon to be safe for field testing with poultry. In addition, the lack of hemolytic activity of 2,2′,4-trihydroxybenzophenone suggests the possibility of designing mechanisms of pharmaceutical availability for production animals both in drinking water and in balanced feed. Doriguetto et al. (2007) when evaluated this same molecule as an anti-inflammatory compound and antioxidant in mice used it orally. The pharmacokinetic properties of 2,2′,4-trihydroxybenzophenone relative to the absorption and permeability estimated from the application of the Lipinski rule says that 2,2′,4-trihydroxybenzophenone presents physicochemical characteristics that are appropriate for good oral bioavailability (Lipinski et al., 2012) and it would be an ideal drug candidate guaranteeing its oral administration.

In the present study, synergism was observed when 2,2′,4-trihydroxybenzophenone was combined with bacitracin, reducing MIC values 4 times (1/4 MIC) for *C. perfringens* ATCC 12924 (Figure 4). Bacitracin interferes with the dephosphorylation of isoprenyl C55-pyrophosphate, which is a molecule that carries the structural elements of peptidoglycans in the bacterial cell wall (Stone and Strominger, 1971). Thus, it is suggested that 2,2′,4-trihydroxybenzophenone acts elsewhere in the bacterial wall resulting in potentiation of the effect of bacitracin.

In Brazil, the use of antimicrobials as growth promoters is allowed. These drugs are used according to the regulations and rules established by the Ministry of Livestock and Supply. In the case of bacitracin, the maximum level to be used in broilers is 50 ppm (MAPA, 2003). The synergism between 2,2′,4-trihydroxybenzophenone and bacitracin decreases the maximum limit suggested by MAPA. This may indicate that besides being a candidate for the treatment of necrotic enteritis in poultry, 2,2′,4-trihydroxybenzophenone may also be used as a growth promoter. Synergism should be considered as an alternative to reduce the dose of bacitracin. This would decrease the chances of acquiring antibiotic resistance, and would also avoid the development of cross-bacterial resistance in humans as reported in several European countries leading to a ban on the use of growth promoters (Charlebois et al., 2012; Han et al., 2015).

2,2′,4-trihydroxybenzophenone can be synthesized in a large scale with an excellent cost-benefit ratio, which may favor its production in comparison with other antimicrobials. It follows the Lipinski’s rule, and presents physicochemical characteristics that are appropriate to a good oral bioavailability and may result in the development of a novel drug.

Thus, 2,2′,4-trihydroxybenzophenone is a potential candidate to be used in poultry farming in the near future. It may be used alone or in association with bacitracin. Further research on the effect of this chemical on naturally infected poultry are required in order to further our understanding on the mechanism of action and effectiveness of this compound in the treatment and prophylaxis of important avian diseases.

## Conclusion

This is the first study assessing the efficacy of the synthetic compound 2,2′,4-trihydroxybenzophenone against Gram-positive and Gram-negative bacteria that cause damage to poultry and public health. Besides, it is also the first study that evaluated the synergistic effect between 2,2′,4-trihydroxybenzophenone and bacitracin. Synthetic 2,2′,4-trihydroxybenzophenone acted on the bacterial cell wall and was non-hemolytic. We conclude that 2,2′,4-trihydroxybenzophenone is a promising antimicrobial candidate that can be used alone or in synergism in research protocols for therapeutic, prophylactic and growth promoting purposes for the poultry industry.

## Author Contributions

MM, MS, and LO conceived the research. MA, GG, and LO performed the research. MB and JP performed the data. MA, GG, MB, and MM wrote the manuscript. All authors approved and contributed to the final version of the manuscript.

## Conflict of Interest Statement

The authors declare that the research was conducted in the absence of any commercial or financial relationships that could be construed as a potential conflict of interest.

## Supplementary Material

FIGURE S1 Antimicrobial activity of 2,2′,4-trihydroxybenzophenone in ATCC bacteria and field isolates. All assays were performed in triplicate and repeated three times. Each point represents the mean value and the standard mean deviation (*P* > 0.05) indicating that there was no significant difference between field isolates and ATCC strains.
