# Supplementary figures and images for: Antimicrobial and Synergistic Activity of 2,2′,4-Trihydroxybenzophenone Against Bacterial Pathogens of Poultry
Source: Front Microbiol. 2019 Mar 20;10:490. doi: 10.3389/fmicb.2019.00490 (PMC6435495; doi:10.3389/fmicb.2019.00490)

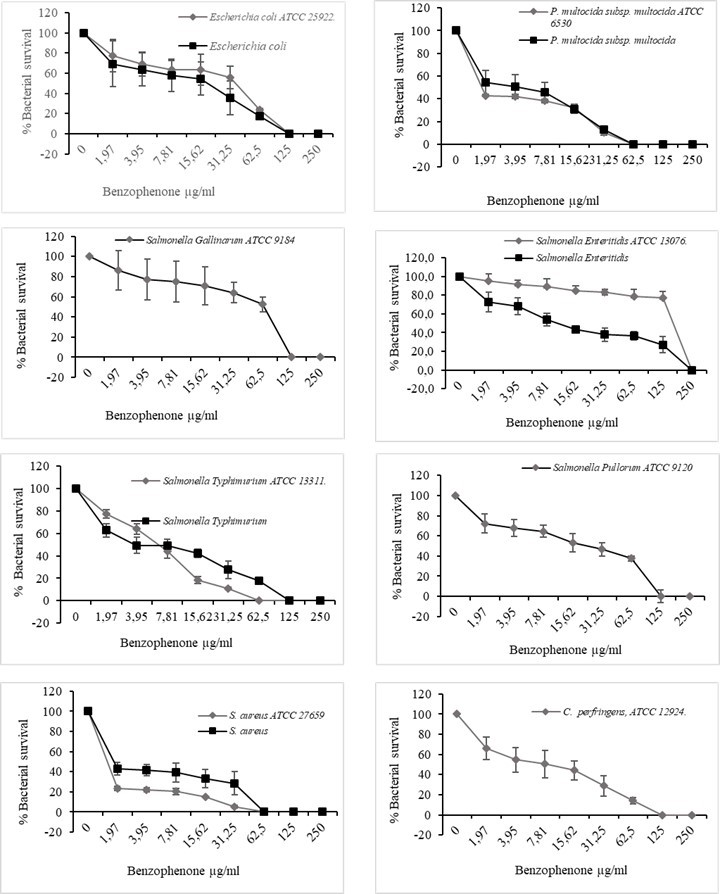

Supplement: FIGURE S1 — Antimicrobial activity of 2,2′,4-trihydroxybenzophenone in ATCC bacteria and field isolates. All assays were performed in triplicate and repeated three times. Each point represents the mean value and the standard mean deviation (P > 0.05) indicating that there was no significant difference between field isolates and ATCC strains. [file Image_1.JPEG]
